# 3-*O*-Ethyl-L-Ascorbic Acid Doped Enteric-Coated Gelatin Capsules towards the Advanced Oral Curcumin Delivery for Cancers

**DOI:** 10.3390/polym14112207

**Published:** 2022-05-29

**Authors:** Dong-Jin Lim

**Affiliations:** Department of Otolaryngology Head & Neck Surgery, University of Alabama at Birmingham, Birmingham, AL 35294-0012, USA; daniel.djlim@gmail.com

**Keywords:** oral drug delivery, cancer prevention, gastrointestinal drug delivery, curcumin, 3-*O*-ethyl-L-ascorbic acid

## Abstract

Among plant-derived polyphenols, curcumin has been recognized as a therapeutically potent nutrient presenting pleiotropic pharmacological effects on various cancers. However, the poor absorption and bioavailability of curcumin limit the use of this excellent naturally occurring polyphenol. 3-*O*-ethyl-L-ascorbic acid (EA) doped enteric-coated gelatin capsules were studied in the search for advanced oral curcumin delivery. The EA doped enteric-coated gelatin capsules were successfully created based on a developed inner dual enteric coating technique. When placed in four buffer solutions with different pHs (pH 2.0, 5.0, 6.0, and 7.3), the coated gelatin capsules showed delayed-release profiles of curcumin below pH 6.0. In contrast, both pristine and fabricated gelatin capsules showed similar curcumin release profiles at pH 7.3, which is a common pH observed in the lower gastrointestinal tract, especially intestinal regions. In conclusion, these results demonstrated the potential of the EA doped enteric-coated gelatin capsules in developing advanced oral delivery of curcumin targeting intestinal-specific regions.

## 1. Introduction

*Curcuma longa* L. (turmeric), one of the ginger family (Zingiberaceae), is a well-known edible plant originally grown in South Asia and India [1]. Most root parts of turmeric, called turmeric rhizomes, are harvested, boiled, and dried to obtain dried turmeric roots, grinding into turmeric powders. In this deep orange-yellow powder, plentiful amounts of curcumin (diferuloylmethane; 1,7-bis(4-hydroxy-3-methoxyphenyl)-1,6-heptadiene-3,5-dione) are present as major polyphenols [2,3]. Turmeric has been widely accepted as a medicinal agent in India, China, and Korea and has been prescribed to treat different diseases such as wounds, arthritis, and skin and eye infections [4]. Interestingly, numerous studies, including recent clinical and mechanistic studies, confirm that curcumin has pleiotropic pharmacological effects. For example, curcumin has antibacterial activity, antioxidant activity, anti-diabetic activity, and anti-inflammatory activity [5,6,7,8,9]. Such diverse benefits in humans are thought to be due to curcumin’s ability to interact with multiple molecular targets, thereby treating numerous indications, including cancers, arthritis, allergies, inflammatory illnesses, and chronic diseases [10]. A variety of molecular targets of curcumin were identified: (1) transcription factors (e.g., NF-κB [nuclear factor-kappa B] and STAT [signal transducers and activators of transcription]), (2) kinases (e.g., EGFRK [epidermal growth factor receptor-kinase] and ERK [extracellular receptor kinase]), (3) receptors (e.g., HER-2 [human epidermal growth factor receptor]) and IL-8R [Interleukin-8 receptor]), and (4) cytokines, enzymes, and growth factors (e.g., MCP [monocyte chemoattractant protein], MIF [migration inhibition protein], iNOS [inducible nitric oxide oxidase], HGF [hepatocyte growth factor], NGF [nerve growth factor], and PDGF [platelet-derived growth factor]) [11]. Cancer has become the most global health burden in the contemporary era, and nearly 19.3 million new cases and 10 million deaths were reported worldwide in 2020 [12]. Paradoxically, even advanced cancer therapeutic options experience the risk of treatment-associated complications and side effects [13]. In particular, long-term use of pharmaceutical anti-cancer drugs is frequently ineffective, leading to cancer recurrence and systemically toxic to other normal tissues [14]. 

In this regard, efforts to use plant-derived supplements or drugs for cancer prevention and treatment have surged. Several anti-cancer drugs derived from plants have already been available on the market, and the discovery of a new cancer drug from plants remains a promising and viable option for cancer [15]. Curcumin has demonstrated meaningful therapeutic activities against bone cancer, melanoma, hematologic cancer (e.g., leukemia and lymphoma), breast cancer, gastrointestinal cancers, head and neck cancer, ovarian cancer, and lung cancer [11,16]. Despite the potential of curcumin in cancer, the poor absorption and bioavailability of curcumin make its use difficult. In a clinical study providing curcumin to patients with advanced pancreatic cancer (8 g per day up to 18 months), only a low nanogram (41 ng/mL) was found in the plasma after six hours [17]. Similarly, a high-dose (10 or 12 g of curcumin powder in a capsule) oral administration to healthy subjects resulted in about 50 ng/mL after four hours, while no curcumin was detected in other relatively low doses (between 0.5 and 8 g) [18]. An incremental dose of curcumin was orally supplemented for patients with pre-malignant or past or ongoing cancer history (e.g., urinary bladder cancer and oral leukoplakia). The average detected levels of curcumin in serum were noted as 0.51 ± 0.11 µM, 0.64 ± 0.06 µM, and 1.77 ± 1.87 µM for 4, 6, and 8 g [19]. Considering the molecular weight of curcumin (368.38 g/mol), these plasma concentrations were equal to 187.87 ± 40.52 ng/mL, 235.76 ± 22.10 ng/mL, and 652.03 ± 688.87 ng/mL, respectively. Such low bioavailability of curcumin has been due to low aqueous solubility, low chemical stability, poor absorption in the gastrointestinal tract (GIT), and rapid metabolism in the GIT and liver [20].

To improve the oral bioavailability of curcumin, many carrier vesicles have extensively been studied. Examples include hydrogel, nanogel, liposome, nano or microemulsion, and nanoparticles [21,22,23,24,25,26]. Not only can advanced micronization techniques, including supercritical antisolvent coprecipitation (SAS), improve the sustained delivery of curcumin, but also the development of an advanced capsule that releases curcumin in a delayed manner can improve the systemic adsorption of curcumin during the travel of GIT [27]. After oral intake, a delayed release of curcumin would help contact the lipophilic molecules to mucus layers covering the intestinal tract, eventually improving the intestinal absorption of this novel but poorly water-soluble polyphenol. A higher concentration of curcumin or curcumin-based formulations can be effectively exposed to the gut through such a delayed delivery system. Curcumin could be packaged into an easy-to-absorption form and absorbed via the enterocytes. Interestingly, an in-vitro permeability study of curcumin using the Caco-2 cell line explained the poor oral availability: (1) instability in the formulation, (2) intercellular accumulation, and (3) intestinal first-pass metabolism [28]. Hence, a well-controlled delivery of curcumin in a therapeutically acceptable concentration without the risk of cytotoxicity in the gut would be an excellent option to increase the reduced bioavailability of curcumin.

**Figure 1:**
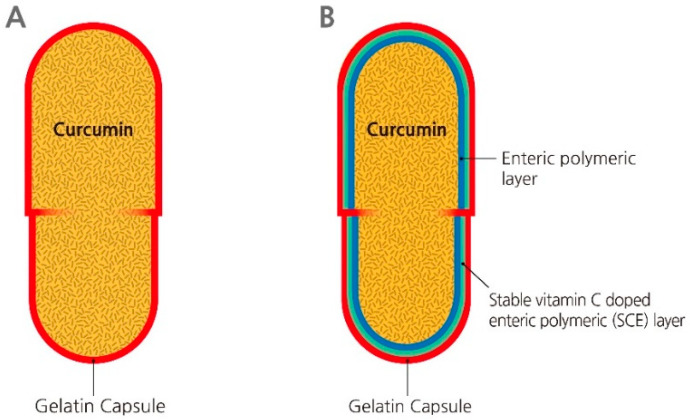
Schematic description of the proposed 3-*O*-ethyl-L-ascorbic acid (EA) doped enteric-coated gelatin capsule. (**A**) a capped gelatin capsule, and (**B**) a capped EA doped enteric-coated gelatin capsule.

In pursuing a reliable but straightforward delayed delivery carrier for curcumin, this study aims to demonstrate the feasibility of an ascorbic acid derivative doped enteric-coated gelatin capsule for releasing curcumin in a delayed manner to the lower gastrointestinal tract, especially intestinal regions (Figure 1). Instead of coating outside a gelatin capsule, an inner dual enteric coating is devised so as not to change the appearance and usability of the gelatin capsule. The inner dual enteric coating technique is expected not only to prevent the fast dissolution of the gelatin capsule but also to achieve a delayed delivery of curcumin at high concentrations in the regions of the intestine. This inner dual enteric coating consists of two distinct polymeric layers: (1) the first inner layer: a stable vitamin C-doped enteric polymer (SCE) layer covering the walls of a gelatin capsule, and (2) the second inner layer: an enteric polymer layer formed after the SCE. For the enteric polymeric coating, an enteric pH-dependent copolymer, called Eudragit^®^ L100-55 (EL 100-55; methacrylic acid-ethyl acrylate copolymer type A, 1:1) is used because this enteric polymer is dissolvable above pH 5.5 aqueous environment. The first SCE layer aims to provide a stable ascorbic acid, 3-*O*-ethyl-L-ascorbic acid (EA), to the second inner layer during the dissolution and disintegration process of the fabricated capsules. Because of the acidity of doped EAs within the enteric polymer of the first inner layer, the first layer continually provides EA to the second enteric polymer, forming a more acidic environment during the disintegration process of gelatin capsules. As a result, the second enteric layer is not easily dissolved even in the elevated pH conditions of GIT. Therefore, the EA doped enteric-coated gelatin capsule can maintain the capsule’s integrity longer than a pristine capsule, thereby delaying the release of the capsule content, curcumin.

To validate this idea, this study is designed as a proof-of-concept study. The objectives of the current study are to demonstrate the potential of the inner dual enteric coating technique in fabricating EA doped enteric-coated gelatin capsules and evaluate the delayed-release profiles of curcumin from the fabricated capsule with different pHs in-vitro. 

## 2. Materials and Methods

### 2.1. Materials 

Curcumin (≥94% curcuminoid purity and ≥80% curcumin) was obtained from Sigma-Aldrich (St. Louis, MO, USA). Gelatin capsules size 4 was purchased from XPRS Nutra (Salt Lake, UT, USA), and 3-*O*-ethyl-L-ascorbic acid (EA) was obtained from TCI America, Inc (Portland, OR, USA). Other chemicals and reagents used in this study were purchased from Thermo Fisher Scientific (Waltham, MA, USA). Eudragit^®^ L100-55 (EL 100-55) was kindly obtained as a gift from Evonik Industries (Essen, Germany)

### 2.2. Coating Procedures 

Two coating steps were performed to obtain gelatin inner dual enteric coatings. For the first inner coating layer, the following procedures were used. After mounting uncapped pristine gelatin capsules (caps and bodies) on a customized try, a 3% (*w*/*v*) EL 100-55 solution containing an additional 3% (*w*/*v*) EA in absolute ethanol was filled and gradually dried under vacuum conditions. When most evaporation occurred, the tray was placed and further lyophilized under a tray-type lyophilizer for 2 days (Advantage Pro, SP Industries, Warminster, PA, USA). Then, the whole tray containing both caps and bodies was placed onto a cabinet desiccator until further coatings. At least 2-days placement was made for complete evaporation of the solvent. In the second coating procedure, a 6% (*w*/*v*) EL 100-55 solution in dichloromethane was refilled into each separated capsule, then placed in a convection oven at room temperature overnight. The dual-coated capsules were lyophilized and placed in a cabinet desiccator until use. 

### 2.3. In-Vitro Release of Curcumin 

**Figure 2:**
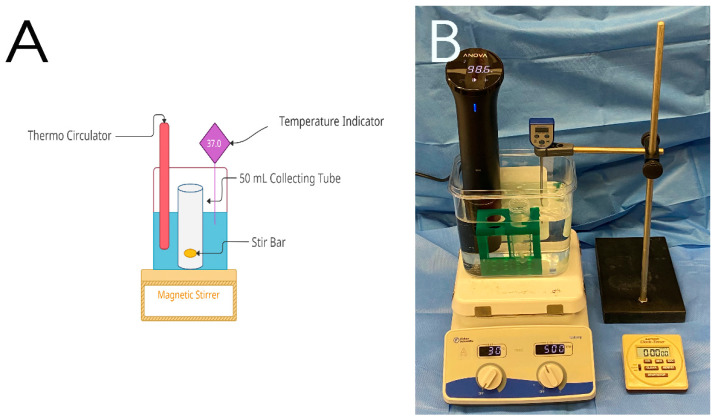
A rapid dissolution apparatus used in this study. (**A**) schematic description of the apparatus and (**B**) the used set-up in this study (37 °C, 500 rpm).

For the curcumin release study, 100 µL of a curcumin nanosuspension (0.5 mg/mL) was immediately loaded into the body of the coated gelatin capsule and assembled into a capsule. The curcumin nanosuspension consisting of sodium alginate, curcumin, polysorbate 80, and calcium chloride was prepared by a slight modification [29]. The curcumin release testing was performed using a rapid dissolution apparatus at 37 °C (Figure 2). In the curcumin release study, four different buffer systems were used: (1) pH 2.0 buffer (0.1 M hydrochloric acid-potassium chloride), (2) pH 5.0 buffer (0.1 M phosphate buffer), (3) pH 6.0 saline (0.9% NaCl), and (4) pH 7.3 HEPES (4-(2-hydroxyethyl)-1-piperazineethanesulfonic acid) buffer. At least three coated capsules were subjected to dissolution study, and the release of curcumin was measured spectrophotometrically. In detail, the curcumin release profiles from the assembled gelatin capsules were obtained by placing at least three capsules in each vessel of the dissolution apparatus and collecting the release media over time. The released curcumin concentration in each sample was evaluated by measuring the absorbance at 422 nm using a microplate reader (Synergy HK, BIO-TEK Instruments, Winooski, VT, USA) [30]. 

### 2.4. Statistical Analysis 

All experiments were performed at least in triplicate. Statistical analysis was performed with GraphPad Prism 6.0 (La Jolla, CA, USA). For comparisons, a paired t-test was performed with a significance set at *p* < 0.05.

## 3. Results 

An ascorbic derivative doped enteric-coated gelatin capsule was fabricated (Figure 1). Ascorbic acid (AA; vitamin C) is an antioxidant involved in enzymatic reactions such as collagen synthesis, carnitine synthesis, and norepinephrine synthesis [31,32,33]. The growing evidence of the role of AA has led to the update of the daily recommended intake from 60 mg to 200 mg per day, as well [34]. Interestingly, this unique but unproducible essential antioxidant contributes to the prevention of causing a disease, which is thought to be partially due to oxidant damage [35,36]. Moreover, AA has been recognized as an anti-cancer therapeutic agent since the inception of an interesting theory of the action of AA on the inhibition of cancer metastasis by William J. McCormick in 1954 [37,38]. In this study, a stable AA derivative, 3-*O*-ethyl-L-ascorbic acid (EA), was used to fabricate the dual enteric-coated gelatins. The reasons were as follows: (1) among several AA derivatives, EA is stable and acidic in aqueous solutions; (2) due to its chemical nature, AA is very unstable in aqueous solutions and the presence of oxygen, even if it is acidic [39]; (3) an enteric polymer EL 100-55 used in this study has a dissolution pH threshold at 5.5, by which EL 100-55 can target the onset of release to the duodenum [40,41]; (4) EA is expected to maintain the pH of the ongoing disintegrated polymeric layer below pH 5.5 when the fabricated gelatin capsules become swollen in the aqueous environment (e.g., saliva and pH 5.5 to 6.5); (5) the delayed-release patterns of this inner dual enteric coating may help develop advanced capsules for the release of curcumin at high concentration in the intestinal tract, thereby improving the therapeutic efficacy of curcumin in the prevention and management of cancers. 

### 3.1. Microscopic Characterization of Pristine Gelatin Capsule

**Figure 3:**
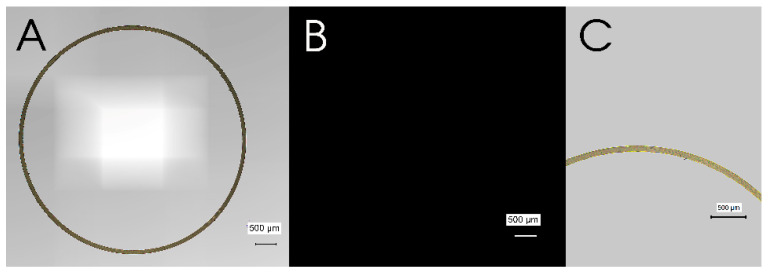
Cross-sectional images of a pristine gelatin capsule. (**A**) a brightfield stitched image, (**B**) a fluorescent stitched image with a red excitation filter, and (**C**) a single brightfield image of the wall of a gelatin capsule. Scale bars indicate 500 µm.

Before performing the coating procedure, the pristine gelatin capsule (size 4) was examined under microscopy. A brightfield stitched image of the cross-section of a pristine gelatin capsule demonstrated the circular shape of the capsules, whereas there is no image appeared when exposed to a red excitation fluorescence filter (Figure 3A,B). The average wall thickness of the used gelatin capsules was 86.8 ± 7.026 µm (n = 5). 

### 3.2. Microscopic Characterization of EA Doped Enteric-Coated Gelatin Capsule 

**Figure 4:**
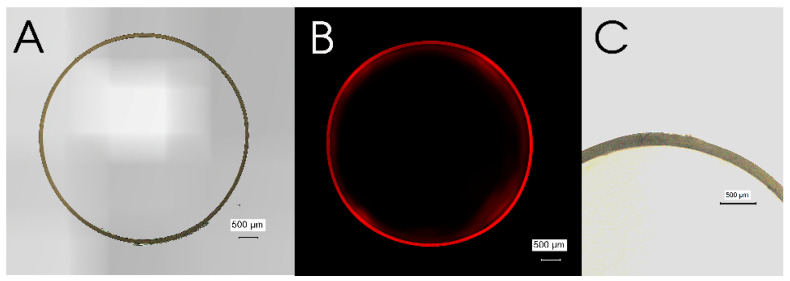
Cross-sectional images of an EA doped enteric-coated gelatin capsule. (**A**) a brightfield stitched image, (**B**) a fluorescent stitched image with a red excitation filter, and (**C**) a single brightfield image of the wall of a gelatin capsule. Scale bars indicate 500 µm.

The fabricated capsules were evaluated by microscopic examinations. To inspect the coating described in the materials and methods section, a fluorescent dye, Nile red, was added in the first coating phase. Like the pristine gelatin capsule, the EA doped enteric-coated gelatin capsule also showed a circular capsule shape under brightfield microscopy (Figure 4A). Moreover, a bright red stitched image was obtained due to the emitted fluorescence of incorporated Nile red, indicating that the coating had been successfully made (Figure 4B). The coating was also confirmed by the changed wall thickness of the capsules. The average wall thickness of the fabricated capsules was 150.0 ± 14.93 µm (n = 5), meaning that the coating thickness on average was 63.2 ± 15.20 µm. 

### 3.3. Release Profiles of Curcumin at Gastric pH (pH 2.0) 

**Figure 5:**
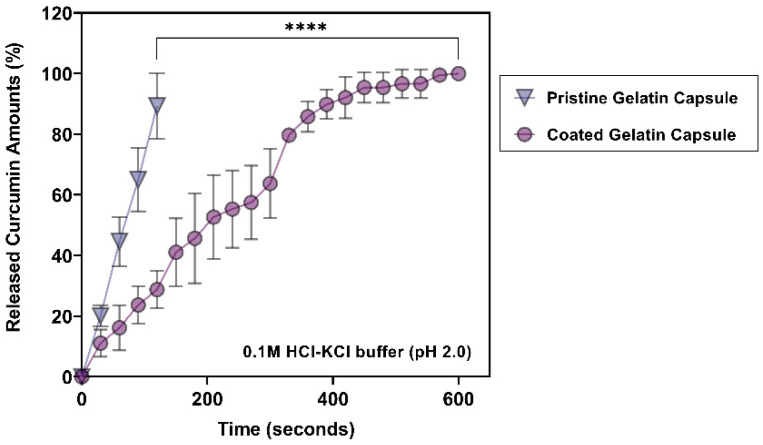
Accumulated curcumin release profiles from different gelatin capsules over time at pH 2.0. Significantly delayed release of curcumin over 10 minutes (600 s) was observed from EA doped enteric-coated gelatin capsules (n = 3 in each condition). ****: *p* < 0.0001.

In a recent study measuring the pH of the GIT by a telemetric swallowable single-use device from twenty healthy subjects, gastric median pH values in fasted-state are reported between pH 1.4 and pH 4.6, and the mean pH value is 2.7 ± 0.8 [42]. In a gastric stimulated buffer (0.1 M HCl-KCl buffer, pH 2.0), the overall release profiles from both groups indicated that the EA doped enteric-coated gelatin capsules showed extended-release of curcumin compared to control gelatin capsules (Figure 5). In detail, half of the loaded curcumin was immediately released from pristine gelatin capsules within 60 s (44.65 ± 8.10%), leading to the complete dissolution of the gelatin capsules after 2 minutes. In contrast, the EA doped enteric-coated gelatin capsules supported the extended-release of curcumin. A relatively extended period (3 minutes) was required to release half of the curcumin (45.65 ± 14.83%), and 10 minutes post-incubation resulted in the complete release of curcumin. The inner coating layers were expected to protect against the rapid disintegration of a gelatin capsule. However, it was difficult to know the role of EA in preventing the capsule’s disintegration in the acidic buffer since EL 100-55 is designed to dissolve only above pH 5.5 aqueous environment.

### 3.4. Release Profiles of Curcumin at pH 5.0

**Figure 6:**
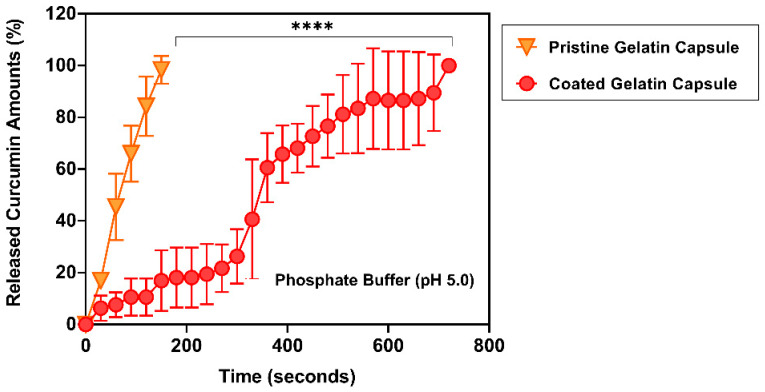
Accumulated curcumin release profiles from different gelatin capsules over time at pH 5.0. Significantly delayed release of curcumin over 12 minutes (720 s) was observed from EA doped enteric-coated gelatin capsules (n = 3 in each condition). ****: *p* < 0.0001.

To evaluate the inner dual enteric-coated gelatin capsules in the gastric condition, both pristine gelatin capsules and EA doped enteric-coated gelatin capsules were placed into a phosphate buffer solution (pH 5.0). The overall release profiles from both groups showed a much more delayed curcumin delivery from the EA doped enteric-coated gelatin capsules (Figure 6). From pristine gelatin capsules, nearly 50% of curcumin was released within 60 s (45.44 ± 12.86%). However, in the EA doped enteric-coated gelatin capsules, a significant period was required to release half of the entrapped curcumin. Almost 6 minutes later, about 60% of curcumin (60.61 ± 13.37%) was released. It was expected that the doped EA from the first coating layer would make the second enteric coating layer acidic when the capsules began to dissolve. Hence, the presence of EA within the fabricated gelatin capsules helps prevent the rapid release of curcumin from the EA doped enteric-coated gelatin capsules.

### 3.5. Release Profiles of Curcumin at pH 6.0

Intraluminal pH changes dramatically between the stomach and the duodenum, where acidic contents originating from the stomach meet a secreted neutralizer (sodium bicarbonate) from the pancreas, liver, and duodenal mucosa in the duodenum [43]. In this region, a gradual increase of the luminal pH is observed: from pH 2.0 to pH 5.0 in the fasting state and from pH 1.7 to pH 4.3 in the second and third postprandial hour [44]. In the fasting condition, the duodenojejunal flexure, located at the border between the duodenum and the jejunum, showed around pH 6.0 (pH 5.98) [44]. For evaluating the pH resistance of the inner dual enteric-coated gelatin capsule in the duodenum, a saline buffer (pH 6.0) was used. 

**Figure 7:**
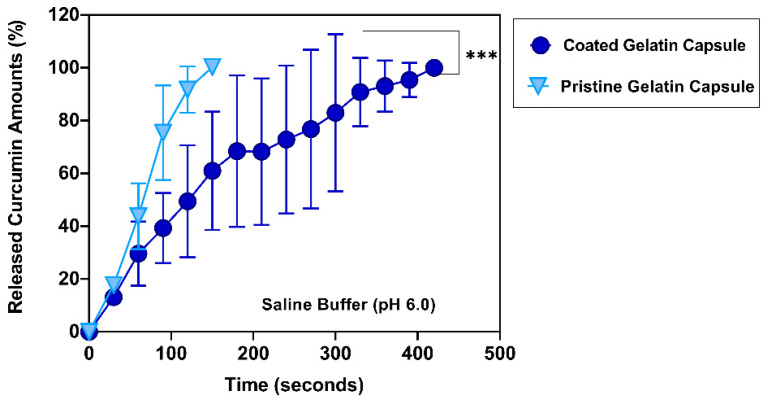
Accumulated curcumin release profiles from different gelatin capsules over time at pH 6.0. Significantly delayed release of curcumin over 7 minutes (420 s) was observed from EA doped enteric-coated gelatin capsules (n = 3 in each condition). ***: *p* < 0.001.

As presented in Figure 7, there was a significantly prolonged release of curcumin from the EA doped enteric-coated gelatin capsules compared to pristine gelatin capsules. The slow dissolution of the fabricated gelatin capsules within a saline buffer showed the delayed-release profiles of curcumin. Like dissolution profiles observed at pH 5.0, the non-coated gelatin capsules needed only 60 s to release half of the curcumin (43.80 ± 12.46%). All curcumin content was released at 150 s with complete disintegration. Whereas the fabricated gelatin capsules kept curcumin inside for a certain period: nearly 50% of the curcumin was released within 120 s (49.48 ± 21.23%); 6 minutes later, more than 90% of the curcumin was released (93.14 ± 9.71%).

### 3.6. Release Profiles of Curcumin at pH 7.3

**Figure 8:**
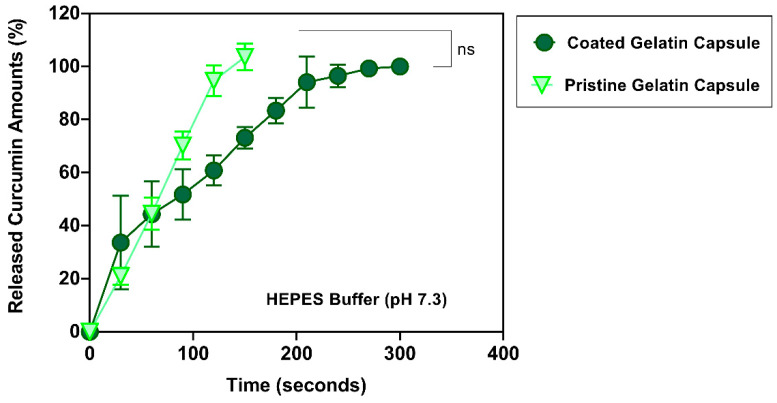
Accumulated curcumin release profiles from different gelatin capsules over time at pH 7.3. (n = 3 in each condition). ns: no statistical difference.

In the small intestine region, slightly alkaline conditions have been reported. For the proximal regions, pH values are around pH 6.0, whereas slightly higher pH values ranging from pH 7.0 to 8.0 have been monitored in the distal areas [42]. In a HEPES buffer solution (pH 7.3), the curcumin release profile from both capsules was similar until 60 s (44.53 ± 6.12% vs. 44.39 ± 12.33%, pristine and coated group, respectively). However, a slightly delayed-release profile was observed in the fabricated gelatin capsule after 60 s, indicating that the doped EA in the first layer of coatings may contribute to the slight disintegration resistance, even in a neutral pH, at which an enteric polymer dissolves. The uncoated capsules disintegrated after 150 s, whereas 300 s was observed for the fabricated gelatin capsules (Figure 8). This delayed disintegration at pH 7.3 was ultimately shorter than the dissolution at pH 5.0 and pH 6.0 (720 s and 420 s, respectively). 

Although there was a slightly delayed profile in the EA doped enteric-coated gelatin capsule, the neutral pH disintegrated both gelatin capsules. These observations were correlated with the molecular characteristics of used EL 100-55 enteric polymers. As noted above, EL 100-55 is a popular enteric coating material for solid forms and is nonionized, exhibiting poor permeability in the stomach’s acidic environment [45]. Because this enteric polymer is designed to dissolve rapidly above pH 5.5, the relative rapid dissolution of both gelatin capsules in a HEPES solution (pH 7.3) was predictable. 

## 4. Discussion and Conclusions

This study is designed as a proof-of-concept, demonstrating the potential of an ascorbic acid derivative-doped enteric gelatin capsule in the targeted delivery of curcumin, which has been reported as a promising naturally occurring polyphenol that prevents and inhibits the progress of cancer development. 3-*O*-ethyl-L-ascorbic acid (EA)-doped enteric-coated gelatin capsules were successfully fabricated using an inner dual enteric coating technique. Then, the release profiles of curcumin in four buffer solutions with different pH values (pH 2.0, 5.0, 6.0, and 7.3) were studied. Overall, the fabricated gelatin capsules exhibited delayed-release profiles of curcumin below pH 6.0 compared to those of typical gelatin capsules. The EA doped enteric coating made from a blend of EA and EL 100-55 made the fabricated gelatin capsules acidic, showing a delayed curcumin release even at pH 6.0. In contrast, the EA doped enteric-coated gelatin capsules showed similar curcumin release profiles to uncoated (pristine) gelatin capsules at pH 7.3, indicating that the fabricated gelatin capsules are liable to a basic physiological condition concerning the pH found in the lower gastrointestinal tract, especially the intestinal regions. There are several limitations to this study. Although the EA doped enteric-coated gelatin capsules showed significantly extended-release of curcumin in all ranges of pH, the retentive time of curcumin is not in the range of reported gastric transit time, which varies between 4 min and 108 min in healthy subjects [46]. However, this study uses a rapid dissolution technique instead of using a conventional certified test protocol to evaluate the potential of the EA doped enteric-coated gelatin capsules. Based on the standardized USP (US Pharmacopeia) 32 chapter <2040> protocol, several studies have indicated that a regular gelatin capsule could release the content over 120 min [47,48,49]. It would be necessary to validate the potential of the EA doped enteric-coated gelatin capsule in providing sustained delivery of curcumin in-vivo studies as well as preclinical studies. Further studies have been planned to characterize the coating efficiency and improve the biocompatibility and safety of the invented coating technique for clinical applications.

In conclusion, the results of this study demonstrate the potential of the ascorbic acid derivative-doped enteric-coated gelatin capsule in pursuing advanced oral delivery of curcumin. Further studies, including in-vivo animal experiments, should be performed to develop EA doped enteric-coated curcumin capsule products for cancer prevention and management.
